# Silencing *GmATG7* Leads to Accelerated Senescence and Enhanced Disease Resistance in Soybean

**DOI:** 10.3390/ijms242216508

**Published:** 2023-11-20

**Authors:** Said M. Hashimi, Min-Jun Huang, Mohammad Q. Amini, Wen-Xu Wang, Tian-Yao Liu, Yu Chen, Li-Na Liao, Hu-Jiao Lan, Jian-Zhong Liu

**Affiliations:** 1 College of Life Sciences, Zhejiang Normal University, Jinhua 321004, China; s.masoud.hashimi@gmail.com (S.M.H.); huangminjun00@163.com (M.-J.H.); q.amini786@gmail.com (M.Q.A.); wangwx@zjnu.edu.cn (W.-X.W.); 19518036769@163.com (T.-Y.L.); ychen10@zjnu.edu.cn (Y.C.); linaliao@zjnu.edu.cn (L.-N.L.); 18659351751@163.com (H.-J.L.); 2 Institute of Genetics and Developmental Biology, Zhejiang Normal University, Jinhua 321004, China; 3 Zhejiang Provincial Key Laboratory of Biotechnology on Specialty Economic Plants, Zhejiang Normal University, Jinhua 321004, China

**Keywords:** autophagy, *Glycine max*, immunity, MAPK, virus-induced gene silencing

## Abstract

Autophagy plays a critical role in nutrient recycling/re-utilizing under nutrient deprivation conditions. However, the role of autophagy in soybeans has not been intensively investigated. In this study, the *Autophay-related gene 7* (*ATG7*) gene in soybeans (referred to as *GmATG7*) was silenced using a virus-induced gene silencing approach mediated by *Bean pod mottle virus* (BPMV). Our results showed that ATG8 proteins were highly accumulated in the dark-treated leaves of the *GmATG7*-silenced plants relative to the vector control leaves (BPMV-0), which is indicative of an impaired autophagy pathway. Consistent with the impaired autophagy, the dark-treated *GmATG7*-silenced leaves displayed an accelerated senescence phenotype, which was not seen on the dark-treated BPMV-0 leaves. In addition, the accumulation levels of both H_2_O_2_ and salicylic acid (SA) were significantly induced in the *GmATG7*-silenced plants compared with the BPMV-0 plants, indicating an activated immunity. Consistently, the *GmATG7*-silenced plants were more resistant against both *Pseudomonas syringae pv. glycinea* (*Psg*) and *Soybean mosaic virus* (SMV) compared with the BPMV-0 plants. However, the activated immunity in the *GmATG7*-silenced plant was not dependent upon the activation of MPK3/MPK6. Collectively, our results demonstrated that the function of *Gm*ATG7 is indispensable for autophagy in soybeans, and the activated immunity in the *GmATG7*-silenced plant is a result of impaired autophagy.

## 1. Introduction

In eukaryotes, macro-autophagy (referred to as autophagy) is a conserved catabolic mechanism that maintains nutrient homeostasis [1]. Under various stress conditions, autophagy mediates the degradation of damaged organelles, protein aggregates that cannot be degraded by 26S proteasome as well as unwanted materials into basic elements in the vacuoles for re-utilization [2,3,4]. In plants, it has been shown that over 40 proteins encoded by autophagy-related genes (ATGs) coordinately participate in the autophagy pathway [2,5]. Autophagy commonly occurs through the formation of double-membrane-bound organelles called autophagosomes that enclose cytoplasmic cargos for autophagic degradation in vacuoles [2]. The formation of autophagosomes starts at a dynamic cup-shaped double membrane structure called the phagophore [6], which is mainly derived from the endoplasmic reticulum (ER) [2,7,8]. After expansion and detachment from the ER, the phagophore surrounds and sequesters damaged or unwanted cytoplasmic materials and organelles followed by fusing and enclosing the double-membraned structure to form an autophagosome [7,8]. The autophagosomes are then delivered to vacuoles, and the outer membranes of the autophagosomes fuse with the tonoplasts to release their contents as autophagic bodies inside vacuoles. The autophagic bodies are degraded to amino acids or other macromolecules and recycled back to the cytosol for re-utilization [2,5,8]. It has been shown that autophagy is involved in numerous biological processes including nutritional starvation responses, growth/development, hormone responses, stress adaptations, senescence, cell death and disease resistance [9,10,11,12,13,14,15,16,17,18].

Autophagy was previously considered a non-selective process. However, it is now apparent that autophagy can be selective to degrade particular cargoes in response to diverse stress conditions [2,15]. Selective autophagy can not only mediate the degradation of ubiquitinated proteins or protein aggregates and damaged organelles but also pathogenic proteins or even entire pathogens [2,18].

Ubiquitin-like Autophagy-related gene 8 (ATG8) mediates selective autophagy. ATG8 protein with lipid phosphatidylethanolamine (PE) attached to its carboxyl terminus (ATG8-PE) is a prerequisite for its anchoring to both the inner and outer membranes of autophagosomes [19]. The C-terminal exposed glycine (Gly) is essential for the formation of ATG8-PE [19]. The exposure of the Gly at the C-terminal of ATG8 is achieved via cleavage by the ATG4 protease from an initially translated longer precursor [2,19].

The formation of ATG8-PE is achieved by a catalytic process similar to ubiquitylation. ATG8 with a Gly exposed at its C terminus is first activated by an ATP-dependent E1-activating enzyme ATG7. The activated ATG7 subsequently binds ATG8 to a conserved cysteine within ATG7 via a thioester linkage. The bound ATG8 is then transferred from ATG7–ATG8 complex to the E2-conjugating enzyme ATG3 by transesterification and ultimately attached to PE catalyzed by an ATG8-specific E3 ligase complex formed by a dimer of the ATG5–ATG12–ATG16 complex [2,19]. Therefore, ATG7, acting as an E1, plays an indispensable role in ATG8–PE formation. The membrane-anchored ATG8s are not only essential for phagophore initiation, elongation, and maturation but also required for selectively recruiting cargoes for autophagic degradation [20,21].

Arabidopsis *atg7* mutants display an accelerated senescence phenotype under normal conditions and exhibit an enhanced sensitivity to carbon and nitrogen starvations [22] as well as many other stress conditions including drought, high salt and oxidative stresses [23,24,25,26]. In addition, autophagic bodies are absent in the vacuoles of *atg7* mutants under autophagy induction conditions [22]. Furthermore, oxidized peroxisomes are over-accumulated in the *atg7* mutant [27,28], suggesting a compromised clearance of damaged organelles. These results demonstrate that the function of ATG7 is indispensable for autophagy. 

A loss in the function of ATG7 results in a reduced resistance to the necrotrophic fungal pathogen *Alternaria brassicicola* [29] but an enhanced resistance to the biotrophic fungal pathogen *Golovinomyces cichoracearum* [30]. Arabidopsis *atg7-2* displays an enhanced sensitivity to iron deficiency [31]. Silencing tomato *SlATG7* reduces the thermotolerance of tomatoes [25]. 

Autophagy has been a hot topic in the past decade and has been extensively studied in the model plant Arabidopsis. However, studies on autophagy in crop plants have just begun. In this study, the function of soybean ATG7 was investigated by the virus-induced silencing approach mediated by *Bean pod mottle virus* (BPMV). Silencing *GmATG7* resulted in an accelerated senescence phenotype under dark treatment, which was accompanied by an over-accumulation of ATG8, indicative of impaired autophagy. In addition, similar to Arabidopsis *atg* mutants, both hydrogen peroxide (H_2_O_2_) and salicylic acid (SA) were significantly accumulated in the *GmATG7*-silenced plants relative to empty vector control plants, indicating an activated defense response. Consistent with this, the *GmATG7*-silenced plants exhibited an enhanced resistance against both *Soybean mosaic virus* (SMV) and *Pseudomonas syringae pv. glycine* (*Psg*) compared with the vector control plants. Lastly, the flg22-induced activation of *Gm*MPK3/6 was reduced in *GmATG7*-silenced plants relative to the control plants, implying that the activated defense responses in the *GmATG7*-silenced plants are independent of *Gm*MPK3/6 activation.

## 2. Results

### 2.1. Silencing GmATG7 Does Not Alter the Morphological Phenotype of Soybean Plants

**Figure 1:**
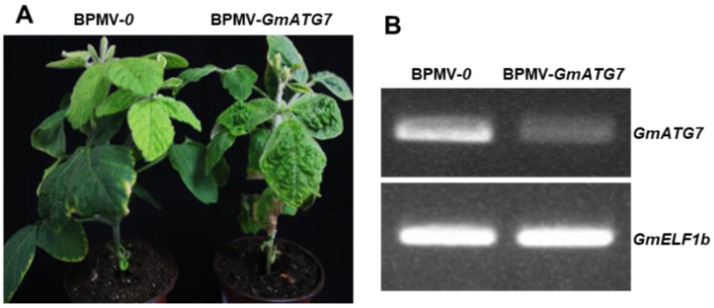
Silencing *GmATG7* did not alter the morphological phenotypes of soybean plants. (**A**) Phenotypes of the vector control plants (BPMV-0) and the *GmATG7*-silenced plants (BPMV-*GmATG7*) under normal growth conditions; (**B**) The verification of *GmATG7* silencing via RT-PCR analysis.

To understand the roles of autophagy in soybeans, a virus-induced gene silencing approach, mediated by the *Bean pod mottle virus* (BPMV), was used to silence multiple *Autophagy-related genes* (*ATGs*), one of which was the *ATG7* homologue. The soybean is a paleotetraploid plant; 75% of the genes in its genome have two copies [32]. However, only one *ATG7* homologue was identified in the soybean genome; therefore, it was referred to as *GmATG7*. A 330-bp fragment of *GmATG7* was cloned into BPMV2 [33,34] and formed the BPMV2-*GmATG7* silencing construct. *GmATG7* silencing was achieved via co-bombardment of BPMV1 and BPMV2-*GmATG7* plasmids coated on gold particles onto two expanded true leaves of 7-day-old soybean seedlings. Meanwhile, BPMV1 and BPMV2 were co-bombarded onto different seedlings and used as empty vector controls (referred to as BPMV-0). At 15–20 days post bombardment (dpb), BPMV symptoms were visible on the upper systemic leaves, indicating successful infection. Systemic leaves with BPMV symptoms were collected and ground into powders with a pestle and mortar and then resuspended into a phosphate buffer (pH, 7.0). After centrifugation, the saps were collected and rub inoculated onto two true leaves of a batch of 7-day-old seedlings. At 15 days post inoculation (dpi), RNA samples were extracted from these plants for the verification of silencing by RT-PCR. After verification, these plants could be used for subsequent experiments. No obvious differences were observed between the *GmATG7*-silenced plants and the empty vector control plants (Figure 1A), indicating that silencing *Gm*ATG7 did not affect growth and development in soybeans. A RT-PCR analysis showed that the *GmATG7* was indeed silenced, as its transcript level was significantly reduced in the *GmATG7*-silenced plants relative to the vector control plants (Figure 1B). 

### 2.2. Silencing GmATG7 Accelerates Leaf Senescence of Soybean Plants under Dark Condition

**Figure 2:**
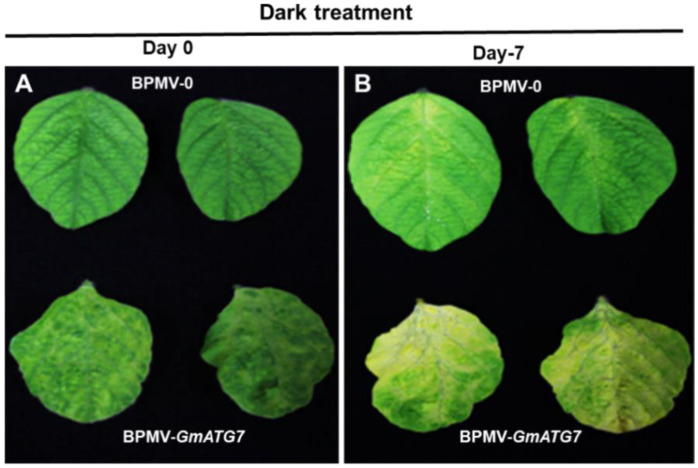
Silencing *GmATG7* resulted in an accelerated senescence phenotype. (**A**) A comparison of leaf phenotypes between BPMV-0 plants and *GmATG7-*silenced plants before dark treatment. (**B**) A comparison of leaf phenotypes between BPMV-0 plants and the *GmATG7-*silenced plants after 7 d of dark treatment.

Under natural conditions, various Arabidopsis *atg* mutants exhibit an accelerated senescence phenotype [30,35,36,37,38,39]. To examine whether the *GmATG7*-silenced soybean plants shared a similar phenotype, the leaves from BPMV-0 and *GmATG7*-silenced plants were collected and dark treated in a Petri dish wrapped with aluminum foil. Long-term dark treatment results in carbon deficiency because of the lack of photosynthesis, which induces autophagy and accelerates senescence. As anticipated, *GmATG7*-silenced leaves displayed a chlorosis phenotype that was not observed in BPMV-0 leaves after the dark treatment for 7 days (Figure 2). This result demonstrated that silencing *GmATG7* leads to autophagy impairment.

### 2.3. Silencing GmATG7 Results in Over-Accumulation of GmATG8 in the Soybean Leaves

**Figure 3:**
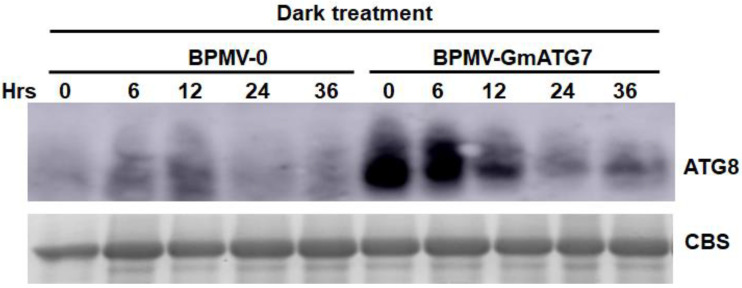
Silencing *GmATG7* resulted in an elevated accumulation level of *Gm*ATG8. A comparison of *Gm*ATG8 protein accumulation level between BPMV-0 control plants and the BPMV-*GmATG7-*silenced plants treated in the dark for different periods of time.

One of the consequences of compromised autophagy is the over-accumulation of ATG8 and other autophagy-related proteins. To test this, Western blotting was performed for the protein samples prepared from dark-treated empty control leaves and *GmATG7*-silenced leaves using an antibody specific to Arabidopsis ATG8, which has been shown to cross-react with the soybean ATG8 [40]. As expected, the accumulation level of *Gm*ATG8 was much higher in the *GmATG7*-silenced leaves than in the empty control leaves (Figure 3), indicating again that silencing *GmATG7* impairs autophagy in soybeans.

### 2.4. Silencing GmATG7 Leads to Over-Accumulation of Both H_2_O_2_ and Salicylic Acid (SA)

**Figure 4:**
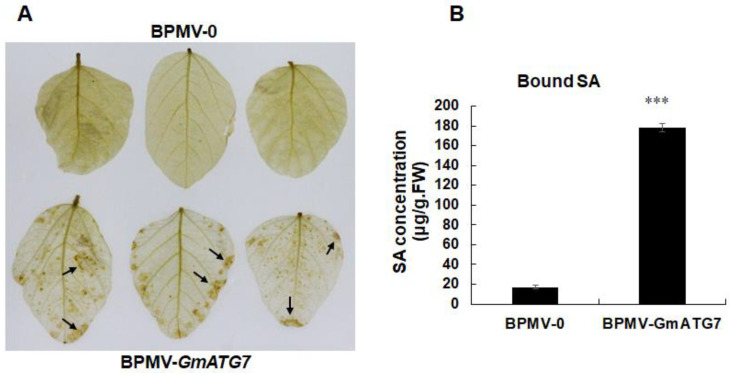
Silencing *GmATG7* leads to the activation of immune responses. (**A**) An elevated accumulation level of H_2_O_2_ was observed in the *GmATG7-*silenced plants via DAB staining. The arrows point to the areas of H_2_O_2_ accumulation; (**B**) Bound SA concentration was significantly higher in the *GmATG7-*silenced plants than in the vector control plants. *** represents *p* < 0.001, Student’s *t*-test. Error bars represent standard deviation (SD).

One of the characteristics of Arabidopsis *atg* mutants is constitutive activated defense responses such as over-accumulation of H_2_O_2_ and SA [30,39,41,42]. To test whether the *GmATG7*-silenced plant shares a similar activated defense response, H_2_O_2_ accumulation was firstly visualized for the dark-treated BPMV-0 leaves and *GmATG7*-silenced leaves using DBA staining. As shown in Figure 4A, the accumulation level of H_2_O_2_ was significantly higher in the leaves of the *GmATG7*-silenced plants than in those of the BPMV-0. In addition, SA accumulation was induced to a much higher level in the leaves of the *GmATG7*-silenced plants than in those of the BPMV-0 (Figure 4B), indicating that silencing *GmATG7* results in constitutive activated defense responses as a result of impaired autophagy.

### 2.5. Silencing GmATG7 Leads to Enhanced Resistance against Soybean mosaic virus (SMV) and Pseudomonas syringae pv. glycinea (Psg)

**Figure 5:**
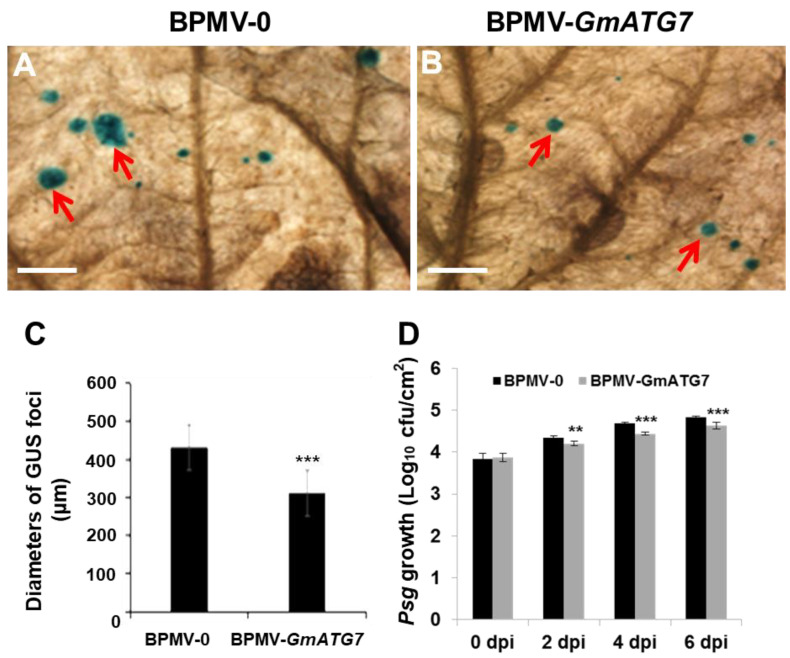
Silencing *GmATG7* leads to the reduced resistance of soybeans to SMV-N-GUS. (**A**,**B**). A comparison of the GUS foci on the leaves of the BPMV-0 (left) and the BPMV-*GmATG7* plants (right) under microscopy; arrows point to the GUS foci; bar = 5 mm; (**C**) A comparison of the diameters of the SMV-N-GUS foci on the leaves of BPMV-0 and BPMV-*GmATG7* plants; error bars indicate that standard deviation was calculated by measuring at least 20 lesions (≥60 lesions) in three independent leaves; (**D**) A growth assay for *Psg* infection. Cfu = colony forming unit. ** represents *p* < 0.01 and *** represents *p* < 0.001, Student’s *t*-test.

The results shown above (Figure 4) demonstrated that silencing *GmATG7* resulted in the activation of defense responses, which are usually positively correlated with enhanced disease resistance. To test the correlation of the activated defense responses observed in the *GmATG7*-silenced plants with disease resistance, we first infected the leaves collected from both the vector control plants and the *GmATG7*-silenced plants with an SMV strain tagged with the GUS reporter gene (SMV-N-GUS) [43] via biolistic particle bombardment. At 3 days post bombardment, the bombarded leaves were incubated with X-Gluc, a substrate of GUS, to visualize the infection foci. A blue GUS foci indicated successful infection, and the diameters of the GUS foci reflect the ability of the replication and cell-to-cell movement of the SMV-N-GUS. As shown in Figure 5A–C, the diameters of the GUS foci on the leaves of the *GmATG7*-silenced plants were much smaller than those on the BPMV-0 leaves, indicating that silencing *GmATG7* leads to an enhanced resistance of soybean plants to SMV.

Next, the BPMV-0 and the *GmATG7*-silenced plants were individually spray-inoculated with *Psg*. The upper and lower ends of the leaves were evenly sprayed with a *Psg* solution. To keep moisture and facilitate infection, all of the plants were then covered with transparent plastic bags after spraying. On different days post inoculation (dpi), leaf discs were collected from the infected leaves for the determination of colony-forming units (cfu). As shown in Figure 5D, the multiplication of *Psg* was significantly lower in the *GmATG7*-silenced leaves than in the BPMV-0 leaves, indicating that silencing *GmATG7* results in an enhanced resistance of soybean plants to *Psg*. Collectively, these results demonstrate that *Gm*ATG7 plays a negative role in soybean immunity.

### 2.6. Silencing GmATG7 Leads to a Reduced Activation of GmMPK3/6 in Response to flg22 Treatment

**Figure 6:**
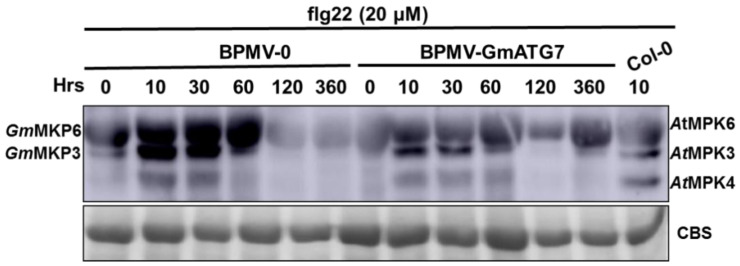
The activation of flg22-induced *Gm*MPK3 and *Gm*MPK6 is significantly reduced in the *GmATG7*-silenced plants. The BPMV-0 and *GmATG7*-silenced plants were treated with 20 μM/L flg22 for the indicated times. Kinase activation was detected via immunoblotting analysis using the phospho-p44/42 MAP Erk1/2 antibody, which specifically recognizes phosphorylated MPK3/4/6. Arabidopsis leaf samples treated with 20 μM/L flg22 for 10 min were used as positive controls. CBS, Coomassie blue staining, was used as a loading control.

It has been previously shown that the MAPK signaling pathway is not affected by the loss of ATG function [29]. To examine the effect of *GmATG7* silencing on the flg22-induced activation of MAPK, the leaves collected from the *GmATG7*-silenced plants as well as from the BPMV-0 plants were treated with 20 μM/L flg22 for 0–360 h. Western blotting was performed on the protein samples prepared from these treated leaves using a p44/42 MAP Erk1/2 antibody, which specifically recognizes the phosphorylated forms of MPK3/6. As shown in Figure 6, flg22-induced activation in the *GmATG7*-silenced leaves was significantly reduced relative to those in the BPMV-0 plants, suggesting that the activated defense responses observed in the *GmATG7*-silenced plants (Figure 4 and Figure 5) is independent of the activation of *Gm*MPK3/6 and *Gm*ATG7 may play a negative role in the flg22-induced activation of *Gm*MPK3/6. 

### 2.7. Silencing GmATG7 Leads to a Reduced Accumulation Level of Ubiquitinated Proteins 

**Figure 7:**
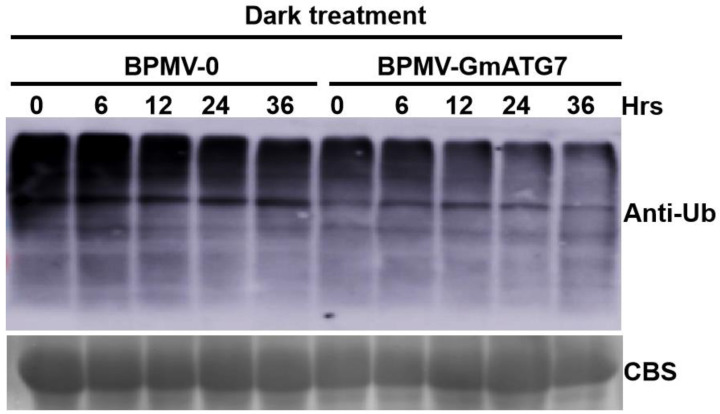
Silencing *GmATG7* genes leads to reduced accumulation of the ubiquitinated proteins under dark treatment. Protein samples were extracted from the *GmATG7-*silenced and the vector plants at 0 min, 6 h, 12 h, 24 h and 36 h post dark treatment. Western blotting was performed by using an antibody raised against the Arabidopsis ubiquitin. CBS staining of the rubisco subunit was used as a loading control (the same as in Figure 3).

Under severe stress conditions, ubiquitinated proteins or protein aggregates, and even the 26S proteasomes, are degraded in vacuoles through the autophagy pathway [44,45]. Ubiquitinated proteins are over-accumulated in various Arabidopsis *atg* mutants due to impaired autophagy [24,46]. Therefore, the accumulation level of ubiquitinated proteins is used as an important indicator to judge whether the autophagy pathway is functional [2,15]. To examine the effect of *GmATG7* silencing on the cellular level of ubiquitinated proteins in soybeans, Western blotting was separately performed for protein samples extracted from the dark-treated leaves of the *GmATG7*-silenced plants and the BPMV-0 plants using an antibody raised against the Arabidopsis ubiquitin. Contrary to our expectations, a significantly reduced level of ubiquitinated proteins was observed in the *GmATG7-*silenced plants relative to the BPMV-0 plants (Figure 7), implying that *Gm*ATG7 plays a negative role in regulating the level of ubiquitinated proteins. However, the molecular mechanism underpinning this phenomenon is not understood. 

## 3. Discussion

The soybean is a paleotetraploid plant, and 75% of the genes in its genome have two copies due to a duplication event [32]. Consistently, all of the genes we previously studied have two copies [47,48,49,50,51,52,53,54]. However, there is only one *ATG7* homolog in soybeans, suggesting that either *GmATG7* was not duplicated, or the other copy was lost after duplication during the course of evolution. As an ATP-dependent E1-activating enzyme, ATG7 plays a critical role in the lipidation of ATG8 (ATG8-PE) and insertion of ATG8-PE on both the outer and inner membranes of autophagosomes [2]. The anchoring of ATG8 to autophagosomal membranes is not only important for the autophagosome biogenesis but also for the selective degradation of proteins that interact with ATG8 through an ATG8-interacting motif (AIM). Selective autophagy is involved in various biological processes such as biotic and abiotic stress responses, drought tolerance and hormone signaling [2,18]. In the absence of ATG7, the lipidation of ATG8 and subsequent recruitment of ATG8-PE to the membranes of autophagosomes is disrupted, leading to impaired autophagy. 

In plants, it has been reported that autophagy plays important roles in nutrient recycling, growth and development, senescence, biotic and abiotic stress responses [2,4,11,15,16,18]. Autophagy has been intensively studied in model plant Arabidopsis. However, it has not been studied in soybeans until recently. In this study, *GmATG7* was successfully silenced in soybeans using a BPMV-VIGS system (Figure 1). Under natural conditions, accelerated senescence and activated defense responses are reported for the Arabidopsis *atg* mutants [30,35,36,37,38,39]. However, the accelerated senescence and activated defense responses were not observed for the *GmATG7*-silenced soybean plants (Figure 1A). This could be due to the fact that the silencing efficacy mediated by the BPMV-VIGS system is not 100%. The silencing efficiency of BPMV-VIGS is usually 80–90% [47]. The remaining unsilenced 10–20% of *Gm*ATG7 transcript in the *GmATG7*-silenced plants is sufficient to maintain the basal level autophagy required under normal growth conditions. However, under autophagy induction conditions, the remaining 10–20% of *Gm*ATG7 is not sufficient to assemble enough autophagosomes. As a result, an accelerated senescence or early chlorosis was observed for the dark-treated leaves of the *GmATG7*-silenced plants (Figure 2).

As an essential part of the autophagosome, ATG8 proteins play critical roles not only in autophagosome biogenesis but also in mediating the selective autophagy for a variety of proteins [2,18]. Along with the autophagosomes, ATG8 proteins are destined to vacuoles and ultimately degraded inside vacuoles [2]. Therefore, ATG8 proteins are over-accumulated in the Arabidopsis *atg* mutants [2]. Consistent with this, *Gm*ATG8 proteins were also over-accumulated in the *GmATG7*-silenced plants (Figure 3), indicating that silencing *GmATG7* indeed resulted in the impairment of the autophagy pathway in soybeans.

The over-accumulation of cellular reactive oxygen species (ROS) and SA is one of the primary reasons of plant senescence and defense responses. One of the functions of autophagy is to maintain the homeostasis of cellular ROS through eliminating damaged organelles such as mitochondria, chloroplasts and peroxisomes, from which the vast majority of stress-induced ROS is derived [18,23,55,56,57]. If autophagy is functional, ROS-induced senescence could be prevented or at least delayed. Concurrently, H_2_O_2_ was over-accumulated in the *GmATG7*-silenced plants (Figure 4), which could explain why accelerated senescence was observed in the *GmATG7*-silenced plants (Figure 2). It has been known for decades that ROS and SA can induce cell death and immune responses and play critical roles in disease resistance [58,59,60]. The activated defense responses observed in the *GmATG7*-silenced plants (Figure 4B) could be a result of the over-accumulation of both ROS and SA (Figure 4A). Consistent with the activated defense responses, *GmATG7*-silenced plants separately exhibited enhanced resistance to the viral pathogen SMV-N-GUS and the bacterial pathogen *Psg* (Figure 5). These results are also consistent with the results from Arabidopsis, which indicated that *atg* mutants display enhanced resistance to biotrophic, bacterial and fungal pathogens. Together, these results suggest that the function of autophagy is conserved between soybeans and Arabidopsis.

The loss function of ATG2 does not have any effect on the activation of MAK/3/6 [29]. Silencing *GmATG2*a*/2b* in soybeans resulted in a reduced activation of *Gm*MPK3/6 induced by flg22 [40]. However, the flg22-induced activation of *Gm*MPK3/6 was elevated in the *GmATG10*a*/10b*-silenced plants [54]. These results indicated that Arabidopsis ATG2 and soybean *Gm*ATG2 have different effects on the activation MAPK signaling pathway, and different soybean ATG proteins have opposite effects on the activation of *Gm*MPK3/6. Similar to *GmATG2*a*/2b* silencing [40], silencing *GmATG7* resulted in reduction in flg22-induced *Gm*MPK3/6 activation (Figure 6), suggesting that *Gm*ATG7 plays a positive regulatory role in *Gm*MPK3/6 activation. Meanwhile, these results also indicated that the enhanced defense responses and elevated disease resistance observed in the *GmATG7*-silenced plants (Figure 4 and Figure 5) are independent of *Gm*MPK3/6 activation.

Under stress conditions, ubiquitinated proteins are eliminated by the autophagy pathway [2,15,18]. Impaired autophagy results in an over-accumulation of ubiquitinated proteins. As a result, ubiquitinated proteins are over-accumulated in Arabidopsis *atg* mutants and *GmATG2a/2b*-silenced soybean plants [24,27,38,40,46,61]. Contrary to this, silencing *GmATG7* resulted in a reduced accumulation of ubiquitinated proteins (Figure 7), suggesting that *Gm*ATG7 might have a role in interfering with the ubiquitination process and thus leads to a reduced level of ubiquitinated proteins. The other possibility is that silencing *GmATG7* could increase the function of the UPS-26S proteasome pathway, either at a transcription level or at a post-translational level, leading to a reduced accumulation of ubiquitinated proteins. The molecular mechanism underpinning this unexpected result remains to be elucidated.

In conclusion, our results demonstrated that the function of *Gm*ATG7 is required for the autophagy pathway in soybeans, and the activated MPK3/MPK6-independent immunity in the *GmATG7*-silenced plants is a result of impaired autophagy. The direction of future work is to reveal which components and signaling pathways are involved in the activated immunity of the *GmATG7*-silenced plants.

## 4. Materials and Methods 

### 4.1. Materials 

#### 4.1.1. Soybean Cultivar

The seeds of the soybeans (*Glycine max* L. ‘Williams 82’) used in this study were harvested from greenhouse-grown plants previously indexed for the absence of BPMV and SMV [47,48]. The soybean plants were grown in a growth chamber at 22 degrees with a photoperiod of 16 h. 

#### 4.1.2. Bacterial Stria (*Escherichia coli*)

DH5α/TOP10 and *Pseudomonas syringae pv*. *glycinea* (*Psg*) R4 strain.

#### 4.1.3. *Soybean mosaic virus* Strain

SMV-N-GUS [43].

### 4.2. flg22 Peptides

The flg22 peptide was purchased from HuaBio (Hangzhou, China). 

### 4.3. BPMV-Mediated VIGS

The BPMV-VIGS system and the inoculation of soybean seedlings with a constructed BPMV-*GmATG7* vector via biolistic particle bombardments using a Biolistic PDS1000/He system (Bio-Rad Laboratories, Hercules, CA, USA) have been previously described [33,34]. The primers used for the construction of the BPMV-*GmATG7* (Glyma.12g010000) are as follows: *GmATG7*-F: **GGATCC**ATAACAGTTCCACAAGGGTG; *ATG7*-R: **GGTACC**CCTGCCTCAATGGGTTAGACAT. The bold sequences are the BamH I and Kpn I restriction sites, which were used for directional cloning. 

### 4.4. RNA Isolation and RT-PCR 

RNA isolation and RT-PCR were performed as described elsewhere. These are the primers used for the verification of the silencing effect: *GmATG7*-V-F: TCACAAGATCCTTTTTGGATTTTA; *GmATG7*-V-R: TCAATTAAATCACACAAACGTTTAC; These are the primers for *GmPR1*: *GmPR1*-F: ATGGGGTTGTGCAAGGTT; *GmPR1-R*: CTAGTAGGGTCTTTGGCCAA; These are the primers used for the reference gene: *GmELF1b*-F: GAGCTATGAATTGCCTGATGG; *GmELF1b*-R: CGTTTCATGAATTCCAGTAGC. 

### 4.5. H_2_O_2_ Detection by DAB Staining 

H_2_O_2_ was detected using the DAB staining method (Sigma-Aldrich, St. Louis, MO, USA), as previously described [62]. 

### 4.6. Inoculation of Pseudomonas syringae pv. glycinea (Psg) 

A *Psg* growth assay was performed as described by [52]. *Psg* was cultivated at 28 degrees to OD = 1.3. The bacterial culture was spun down, and the pellet was re-suspended in an LB medium with an OD = 1. The vector control or *GmATG7*-silenced plants were sprayed with the re-suspended bacterial solution on both sides. The sprayed plants were immediately covered with transparent plastic bags to maintain moisture and facilitate infection. 

### 4.7. SMV-N-GUS Inoculation, GUS Staining, and GUS Foci Measurements 

At 18 dpi infected with only the BPMV vector (BPMV-0) or BPMV-*GmATG7* constructs, a second fully expanded soybean trifoliolate leaf, counted from the top, was detached and biolistically inoculated with SMV-N-GUS [51]. Following SMV-N-GUS inoculation, the detached leaves were put into Petri dishes with moist filter papers and kept on a lit growth shelf for 5 days before GUS staining. GUS staining was performed as described by [63]. Photographs of the leaves with GUS foci were taken using a stereo microscope (Olympus SZH10, Center Valley, PA, USA). The numbers of GUS foci were counted, and the diameters of GUS foci were measured using Soft Image System analysis (IA Package; Olympus). 

### 4.8. Western Blotting Analysis

Proteins were extracted from dark-treated BPMV-0 and BPMV-*GmATG7* leaves. Western blotting was performed using Anti-Ubq (Agrisera, AS08307, Vännäs, Sweden) or anti-ATG8 (Agrisera, AS142769), as previously described [51]. 

### 4.9. GmMPK3/GmMPK6 Kinase Activation Assay 

Protein samples were extracted from leaf tissues of indicated soybean plants in the extraction buffer (50 mM Tris-MES pH 8.0, 0.5 M sucrose, 1 mM MgCl_2_, 10 mM EDTA, 5 mM DTT) in the presence of protease inhibitor cocktail S8830 (Sigma-Aldrich, St. Louis, MO, USA), as described [51]. The protein extracts were spun at 12,000 rpm at 4 °C for 30 min, and the supernatants were collected for Western blotting analysis. Protein separation was conducted using SDS-PAGE; then they were transferred to a PVDF membrane (Millipore, Billerica, MA, USA) via semi-dry electro-transfer (Bio-Rad, Hercules, CA, USA) in accordance with the manufacturer’s manual. The membrane was blocked for 2 h in a 1× TBST buffer containing 5% milk powder. After washing 3 times, the membrane was incubated with human-derived anti–Phospho-p44/p42 MAPK (anti-pTEpY), which specifically recognizes phosphorylated MPK3/MPK4/MPK6 across kingdoms, and then diluted at 1:3000 (Cell Signaling Technology, Danvers, MA, USA), followed by incubation with a secondary antibody. The bands were visualized via incubation with a chemiluminescent HRP substrate (Millipore, Billerica, MA, USA). 

### 4.10. Statistical Analysis 

Each experiment was repeated three times, and the representative results are presented. Statistical analyses were performed using SPSS Statistics 27 (Statistical Product and Service Solutions, IBM, Armonk, NY, USA). The test methods are indicated in the figure legends. 

## Data Availability

Data are contained within the article.
